# Statin‐associated myopathy. Assessment of frequency based on data of all statutory health insurance funds in Germany

**DOI:** 10.1002/prp2.404

**Published:** 2018-05-10

**Authors:** Peter Ihle, Franz‐Werner Dippel, Ingrid Schubert

**Affiliations:** ^1^ PMV forschungsgruppe Department of Child and Adolescent Psychiatry, Psychosomatic and Psychotherapy University of Cologne Cologne Germany; ^2^ Sanofi‐Aventis Deutschland GmbH Berlin Germany

**Keywords:** claims data, myopathy, pharmacoepidemiology, statin intolerance

## Abstract

Aim of the study was to assess the incidence of statin‐associated myopathy (SAM) under real‐life conditions in Germany. Database: Administrative data (master data, diagnoses, prescriptions) for all individuals in Germany insured with the Statutory Health Insurance. Basic population: individuals 18 years and older who have been insured continually from 2009 to 2011 (52.9 million; 29.9 million men, 23.9 million women). Data access is provided by the German Institute of Medical Documentation and Information, DIMDI) according to the Data Transparency Regulation of 2012. Statins: identification with the ATC–Codes: C10AA, C10BA and C10BX. Study population: incident statin users in 2010 with a diagnosis of lipid disorders (ICD‐10‐GM E78, excluding patients with: E78.1, E78.3, E78.6 in eight quarters before index prescription. Definition of SAM: documentation of myopathy (ICD‐10‐GM G72.0, G72.8; G72.9, M60.8, M60.9, M79.1) in the first statin prescription quarter or in one of the three following quarters. The first event is considered for the incidence estimate. The daily doses included in a package were classified as “days under therapy” (by assuming one DDD) and taken as exposition time. SAM was found in 1.9% of 531 672 incident statin users. The percentage differs according to the patterns of statin use: the lowest incidence is observed in those with only one prescription (1.3%), the highest incidence with 5.0% is observed in those who not only stopped the treatment within 365 days, but who also had their statin changed. Administrative data including diagnoses from ambulatory care provide a realistic estimate of SAM frequency in every day practice.

**Abbreviation**
ATC: Anatomical Therapeutic Chemical (ATC) classification system
CK: creatine kinase
CTT: Cholesterol Treatment Trialists
DaTraV: Information system health care data
DDD: Defined Daily Dose
DIMDI: German Institute of Medical Documentation and Information
ICD 10 GM: International classification of Diseases‐ German Modification
PAD: peripheral arterial disease
SAM: statin associated myopathy
SHI: statutory health insurance
WHO: Word Health Organisation

## 1 INTRODUCTION

Statin therapy has great importance in the treatment of lipid metabolic disorders and in the prevention of cardiovascular events and therefore is recommended as the therapy of first choice in international and domestic guidelines, along with lifestyle changes.^1^, ^2^, ^3^, ^4^ Reduction in morbidity and mortality in secondary prevention is well supported by numerous studies as well as meta‐analyses.^5^, ^6^, ^7^, ^8^ The implementation of the therapy recommendations is seen not least in the prevalence of treatment with statins, which has been increasing for years.^9^, ^10^ In Germany, lipid‐lowering agents are reimbursed by the statutory health insurance if a cardiovascular disease (coronary heart disease), cerebrovascular disease (stroke) or peripheral arterial disease (PAD) is manifest or there is a high cardiovascular risk with an estimated probability of occurrence ≥20% in the next 10 years.^11^ Current US guidelines recommend increased use even in primary prevention.^12^ Against the backdrop of treatment rates, which are already high and are expected to continue to increase, reliable information is needed not only about benefits, but also about possible harm from the therapy, such as increased risk of diabetes, disorders of liver and kidney functions, muscle damage, as well as the frequent occurrence of malignant diseases.^4^ Here, the evidence is clearly more uncertain compared to the benefits. Since cerivastatin had to be withdrawn from the market in 2001 due to cases of lethal rhabdomyolysis, muscle damage and muscle ailments have been given greater attention as a possible side effect of the statin treatment. Nonetheless, in Germany, there is a lack of reliable data, such as how frequently muscle disorders and muscle ailments occur. The frequency estimates of statin intolerance are scattered in the literature depending on the definition and investigation method, from 0.01% to 10%.^13^ Registries and observational studies identified values from 7% to 29%.^14^, ^15^ In clinical trials (RCTs), in contrast, there are only minor differences in the side effects compared to placebo.^16^ However, experience in everyday health care seems to be different—a difference that has been seen not least in the controversy between the Cholesterol Treatment Trialists (CTT) Collaboration and the British Medical Journal 17, 18, 19 since 2014 and which raises the question of whether fewer severe side effects in clinical trials are possibly under‐reported because of methodological reasons.^20^, ^21^

Statin treatment generally represents long‐term therapy, but there are strong indications of insufficient adherence and premature discontinuation of treatment.^22^, ^23^ Risk for non‐adherence is higher in males and increases with age, comorbidities like anxiety and depression and also with reported bad news about statins 24, 25, 26 Discontinuation rates in everyday care due to side effects are higher than the rates of side effects described in clinical studies. One reason for discontinuation of therapy or for therapy interruptions may be the occurrence of muscle pain. Studies show that patients with statin intolerance have higher rates of cardiovascular events.^27^, ^28^

Since there is no information for Germany on the frequency of statin‐associated myopathy (SAM) under everyday conditions, the goal of the study was to develop a method to report SAM based on routine data from the statutory health insurers and to make an estimate of the frequency of statin‐induced myopathy based on that data. There are different definitions of SAM in the literature.^21^, ^29^ In the following, we generally refer to any kind of muscle‐related complaints with and without CK elevation. Statin‐associated does not necessarily mean that there must be causality.^29^

## 2 MATERIALS AND METHODS

### 2.1 Database

The analysis was conducted on a Germany‐wide statutory health insurance (SHI) data set, the so‐called “Health Care Data Information System”, which is located at the German Institute of Medical Documentation and Information (*Deutsches Institut für Medizinische Dokumentation und Information,* DIMDI) (hereinafter referred to as DaTraV data). This database of approx. 70 million SHI insured persons out of 80.8 million inhabitants, which was established based on a statutory provision, has been available since February 2014.^30^ The basis is data that all statutory health insurers have to transmit for the morbidity‐oriented risk structure compensation scheme to the authority responsible for this, the Federal Social Insurance Authority (*Bundesversicherungsamt*, BVA). The DIMDI is receiving this insured person‐based pseudonymized data with sociodemographic information on the insured persons (birth year, gender, insurance days) and information on the diagnoses coded in the outpatient and inpatient sector (according to ICD 10‐GM) as well as the drugs dispensed in the outpatient sector and reimbursed by the SHI. For analyses of this database, an SQL analysis script was submitted on 3 August 2015. Before the result sets are transmitted, an intensive review is conducted by the DIMDI to determine whether the insured person's identity is protected. A cell population of at least n = 30 is required for this. It is also reviewed whether, as a result of dependencies within the results table or even due to dependencies between the results tables, for example, through subtraction or comparisons of marginal totals, cell populations below the above‐cited minimum case number of n = 30 can also be approximately calculated. After repeated aggregation of the results tables by the applicant and intensive review by the DIMDI, the result sets were transmitted in compliance with data privacy law on 22 Dec. 2016).

At the time of the analysis, prescription data as well as inpatient and outpatient diagnostic data were available from the reporting years 2008 to 2011. There are no performance figures available from which a measurement of creatine kinase (CK) could be made. For the inpatient stays, it is only documented in which month the hospital stay took place.

### 2.2 Statin prescriptions

These are calculated in the data set based on the Anatomic‐Therapeutic‐Chemical‐Classification (ATC code) C10AA for monosubstances and C10BA as well as C10BX for combinations (ATC Version 2012).^31^

### 2.3 Study population

The population consists of all continuously insured persons of the SHI for the period 2009 to 2011 who are 18 years and older (52.9 million persons, of whom 29.0 million are men and 23.9 million women). The study population is incidental statin recipients from 2010 with documented lipid metabolic disorder. A statin recipient was defined as incidental if in the data set, before the first statin prescription in 2010, no statin prescription was documented in 2008 and 2009. This means a therapy‐free period of at least 2 years.

#### 2.3.1 Inclusion and Exclusion Criteria

The presence of a lipid metabolic disorder was assumed if one of the following diagnoses‐according to ICD 10‐GM 32‐was documented in the lead time or in the incidence quarter at least once in the outpatient sector (with modificator “confirmed” to indicate the diagnostic confidence) or as a hospital diagnosis: E78.0: Pure hypercholesterolemia, E78.2: Mixed hyperlipidaemia, E78.4: Other hyperlipidaemia, E78.5: Hyperlipidaemia, unspecified, E78.8: Other disorders of lipoprotein metabolism, E78.9: Disorder of lipoprotein metabolism, unspecified. Insured persons with the following diagnoses were explicitly excluded: E78.1: Pure hypertriglyceridaemia, E78.3: Hyperchylomicronaemia and E78.6: Lipoprotein deficiency in the prior eight quarters. Likewise, insured persons were excluded for whom muscle pain had already been coded in the eight quarters before the first prescription quarter with a statin (see further below). These diagnoses had to have been documented either once as an outpatient (with the modifier “confirmed”) or as an inpatient diagnosis. Outpatient diagnoses are documented only quarterly, inpatient discharge diagnoses at precise monthly intervals.

### 2.4 Definition of statin‐associated myopathy (SAM)

Since there is no specific coding for this, SAM must be determined indirectly from the SHI data. We assume SAM in cases where SAM is documented for the first time in new users of statins (in the prescription quarter or in one of the three following quarters) and where no documentation of SAM is found during the eight quarters before starting statins. Myopathies that were documented after the end of the statin therapy were not included in the analysis. Muscle pain was recorded here with following ICD 10‐GM‐coded diagnoses: G72.0: Drug‐induced myopathy, G72.8: Other specified myopathies, G72.9: Myopathy, unspecified, M60.8: Other myositis, M60.9: Myositis, unspecified, M79.1: Myalgia. The WHO Code M62.82 Rhabdomyolysis is not available in the German Modification. In each case, the first event with exposure is considered.

### 2.5 Statin utilization pattern and SAM

Estimation of the SAM requires that the exposure time, ie, the period of treatment with statins, be determined for every statin recipient. In the SHI routine data, there is no information on the prescribed dose from which the range of coverage of the prescribed package could be determined. For this reason, the daily doses included in a package were classified for this as “days under therapy”. This approach was established as a quasi‐standard for the secondary data analysis of SHI routine data.

In the case of insured persons who are given a lower dose than one daily dose per day and for whom, consequently, the range of coverage of the package would be longer, when a DDD is used to calculate the range of coverage of the package, there are interruptions in therapy due to methodological reasons. This calculative therapeutic gap was “closed” if the number of days was smaller than the range of coverage of the last package. In terms of the calculation, this means a doubling of the range of coverage and thus halving the daily dose for the last prescribed package before the interruption. If the therapy gap, in contrast, was longer than the number of daily doses included in the package, only the single daily dose was taken as the range of coverage, and an interruption in therapy was identified. For the calculation of the range of coverage, combination drugs had to be recoded into the individual active substances, for example, the combination drug of simvastatin and ezetimibe (ATC code C10BA02) into simvastatin C10AA01 and ezetimibe ATC code C10AX09. For the calculation of the duration of therapy, the daily dose of the combination drug was transferred to both individual substances. If the combination drug contained 50 daily doses, for example, then each of the two monosubstance shares was also entered with 50 daily doses into the calculation of the duration of the range of coverage.

Based on the pattern of use, different populations can be seen with respect to (i) the duration of therapy (end of therapy within 364 days), (ii) the continuity of treatment (no follow‐up prescription after a gap according to the number of daily doses of the last prescription) as well as (iii) with respect to the occurrence of a change in the statin.

The frequency of SAM is determined for all insured persons with incidental statin prescription as well as for populations that are differentiated by the (i) duration of the statin treatment, (ii) continuity of the therapy and (iii) by a change in the statin therapy. In an additional analysis, the share with SAM is determined for the subgroup of incidental statin recipients in whom a change in the statin with subsequent discontinuation of therapy could be observed. Included here were insured persons with first‐time statin therapy with the following active substances (all strengths): Atorvastatin, fluvastatin, lovastatin, pitavastatin, pravastatin, rosuvastatin, simvastatin in which a change to a second statin as well as a therapy discontinuation of the second statin and no additional statin therapy were observed within the investigation period of 365 days after the index date.

### 2.6 Statistics

Frequency estimates are made in percentage values. The calculation was made with SQL Server 2016 under Windows Server 2016.

## 3 RESULTS

Using the definition for the study population, results in 531 672 incidental statin recipients with a lipid metabolic disorder in 2010 (cf. Table 1). Based on the continuously insured persons as the base population in this year, the share is around 1%.

**Table 1:** Incidental statin recipients and percentage of the SHI insured population in Germany, 2010

Age group	Men		Women		Total	
	N	%	N	%	N	%
18‐29	1112	0.03	987	0.03	2099	0.03
30‐39	3476	0.09	6817	0.20	10 293	0.14
40‐49	18 229	0.32	34 012	0.68	52 241	0.49
50‐59	54 308	1.10	61 777	1.42	116 085	1.25
60‐69	74 571	1.88	68 992	2.05	143 563	1.96
70‐79	84 918	2.15	66 511	2.24	151 429	2.19
80‐89	35 078	1.74	17 794	1.92	52 872	1.80
>= 90	2406	0.82	684	1.01	3090	0.85
Total	274 098	0.94	257 574	1.08	531 672	1.01

Database: Information system health care data (DaTraV) according to Social Insurance Code (SGB) V §303a‐e, here: Continuously insured persons from 2008 to 2011, 18 years and older.

The statin recipients are described in the following initially under the three aspects: (i) Duration of prescription, (ii) Continuity of treatment and (iii) Type of therapy, ie, with or without change in the statin. In the next step, the analysis of the documentation of an SAM is made overall (according to the type of SAM) as well as according to the previously described prescription pattern.

The analysis of the prescription duration shows that roughly one quarter of the statin recipients received only a one‐time prescription without a follow‐up prescription in the course of the first 12 months after an incidental statin prescription. In another quarter, the therapy was ended within 364 days. Just under half of the statin recipients (49.1%), in contrast, were still treated after 365 days; 33.6% were treated continuously for 1 year. A change in statin was observed in around 14 000 incidental statin recipients in the period of 364 days after the first prescription, with such a change occurring already in more than one‐third of the statin recipients within the first 91 days. In the last quarter, a statin change was observed only in just under 18%.

Table 2 shows the results of the duration of therapy and continuity together with the indication of whether different statins were prescribed, ie, a “change of active substances” occurred.

**Table 2:** Duration of prescription starting with the incidental statin prescription according to continuity and change in therapy

Duration of therapy	Treated continuously	Incidental statin recipients, of them with change		
		N	N	%
End of therapy within 364 d	No	71 847	4708	6.6
	Yes	63 949	1634	2.6
Statin therapy until end of the year	No	82 815	5414	6.5
	Yes	178 383	2531	1.4
Total (without one‐time prescription)		396 994	14 287	3.6

Database: Information system health care data (DaTraV) according to Social Insurance Code (SGB) V §303a‐e, here: incidental statin recipients from 2010.

### 3.1 Frequency of Statin‐associated Myopathy (SAM)

In the 1‐year follow‐up period of 531 672 incidental statin recipients, myopathy according to the included diagnoses was documented in 10 250 persons (1.93%). Myalgia (ICD 10‐GM: M79.1) was documented in almost 94% of the cases (cf. Table 3). The specific diagnosis of “drug‐induced myopathy” was given for only 58 recipients, equivalent to a share of 0.6%.

**Table 3:** Insured persons with statin intolerance according to the type of myopathy diagnosis

ICD 10 GM Code	Name	Patients with SAM	
		N	%
G72.0	Drug‐induced myopathy	58	0.6
G72.8	Other specified myopathies	79	0.8
G72.9	Myopathy, unspecified	325	3.2
M60.8	Other myositis	43	0.4
M60.9	Myositis, unspecified	255	2.5
M79.1	Myalgia	9598	93.6
Sum		10 250	100.0

Database Information system health care data (DaTraV) according to Social Insurance Code (SGB) V §303a‐e, here: incidental statin recipients from 2010.

Viewed over time, the myopathy diagnosis was documented primarily in the first quarter of treatment (cf. Table 4).

**Table 4:** Distribution of myopathy diagnoses over time

In the quarter according to incidence	Patients with statins and SAM	
	N	%
1	4498	43.9
2	2117	20.7
3	1684	16.4
4	1951	19.0
Total	10 250	100.0

Database: Information system health care data (DaTraV) according to Social Insurance Code (SGB) V §303a‐e, here: incidental statin recipients from 2010 with myopathy diagnosis.

Table 5 shows the incidental statin recipients in relation to the continuity of therapy, the change and the documentation of a myopathy diagnosis in every therapy group. The share with SAM is lowest in the group of insured persons with one‐time prescription (1.3%) and highest with 4.8% of the group who had ended their statin therapy already before the end of the year and had an interruption and a change.

**Table 5:** Statin‐associated myopathy (SAM) with categorization of incidental statin recipients and therapy end, interruptions and changes

Duration of therapy	Treated continuously	With change in statin	Incidental statin recipients		
			N	Of them with statin intolerance	
				N	%
One‐time prescription			134 678	1770	1.3
End of therapy within 364 d	No	No	67 139	1366	2.0
	No	Yes	4708	225	4.8
	Yes	No	62 315	1288	2.1
	Yes	Yes	1634	81	5.0
Statin therapy until end of the year	No	No	77 401	1755	2.3
	No	Yes	5414	224	4.1
	Yes	No	175 852	3440	2.0
	Yes	Yes	2531	101	4.0
Total			531 672	10 250	1.9

Database: Information system health care (DaTraV) data according to Social Insurance Code SGB V §303a‐e, here: incidental statin recipients from 2010.

It is clear from Table 6 that statin recipients with documentation of a statin change have a higher share with SAM than recipients without such a change. If the statin recipients are summarized according to occurrence or non‐occurrence of a change, in the first group, with 4.4%, the share with SAM is more than double that of the group of non‐changers (2.1%) (cf. Table 6). This could be interpreted as an indication that a change in the active substance was made here due to complaints. Since the documentation of the outpatient diagnoses is made only on a quarterly basis, it cannot be shown whether the SAM diagnosis was documented before or on the day of prescription of another statin.

**Table 6:** Statin‐associated myopathy (SAM) with categorization of incidental statin recipients according to type of therapy and change

Duration of therapy	Incidental statin recipients			
	N	%	Of them with SAM	
			N	%
One‐time prescription (no statin change possible)	134 678	25.3	1770	1.3
At least 2 prescriptions without statin change	382 707	72.0	7849	2.1
2 prescriptions with statin change	14 287	2.7	631	4.4
Total	531 672	100.0	10 250	1.9

Database: Information system health care data (DaTraV) according to Social Insurance Code (SGB) V §303a‐e, here: incidental statin recipients from 2010.

A change in the statin with subsequent discontinuation of therapy occurred in 2010 in 10 056 persons; a SAM diagnosis was coded in 416, equivalent to a share of 4.14%.

## 4 DISCUSSION

Based on a Germany‐wide data set spanning all statutory health insurance fund types, a frequency of statin‐associated myopathy of 1.9% was identified in statin recipients in the first year after the start of therapy, based on the diagnosis documentation. To our knowledge, this was the first estimation of the frequency of SAM conducted for Germany on the basis of routine data. The identified SAM rate, at 2%, differs substantially between patients who are treated without observable interruptions in therapy and statin changes and patients whose therapy ended within the first treatment year and at least one statin change (5%). Consistent with other authors,^29^, ^33^ we also see that statin therapies are continued after a change in the active substance.

With respect to patients who were given continuous treatment without statin changes and were diagnosed with SAM, our interpretation is that the muscle pain occurring under therapy was discussed and documented in the doctor‐patient contact in the outpatient sector. A dose reduction might have been made. However, this was not able to be investigated using the available database.

If the different diagnoses that are subsumed under SAM are broken down individually, at first glance, it is confusing that “drug‐induced myopathy” is named as a diagnosis, and also that unspecific diagnoses are supplemented with the description “unspecified”. However, in Germany, this is consistent with the general diagnosis coding behavior in the outpatient sector. Here, the emphasis is on continued treatment and not the clarification of possible causality.

Thus, just as there are different definitions for the diagnosis of statin intolerance and SAM,^21^, ^34^, ^35^, ^36^ the studies also differ in their methodological approaches to reporting the incidence rate. Our estimate, for example, is lower compared to the information from practitioners on the frequency of statin‐associated symptoms in their patients, whom Hovingh et al. 33 identified in 13 countries with a survey. According to the assessment of treatment providers, in Germany, 71% of incidental statin recipients complain of muscle pain, 4% cannot tolerate the recommended dose. Of them, according to an estimate, 64% (equivalent to 2.5% of incidental statin recipients) have SAM. However, a comparison of the results is possible only to a limited extent due to the different methodologies (survey vs routine data).

There are some international studies that address estimating the frequency of SAM using administrative data,^26^, ^37^, ^38^, ^39^, ^40^, ^41^ but they consider either only severe SAM based on hospital data (hospitalization due to SAM) or SAM restricted to rhabdomyolysis 42 and/or include laboratory values, and thus from a methodological standpoint are not comparable with the study conducted here, which observes SAM exclusively by means of diagnoses (including in the outpatient sector). Therefore, it may be assumed that in these hospital‐based studies, only the most severe SAM is recorded, and the estimates of incidence rates are accordingly lower.

Chang et al. 43 also consider, in their analysis based on claims data, diagnoses from the outpatient sector, but calculate these from the plain text (myositis or rhabdomyolysis). Among 18 036 incidental statin recipients, they identified 23 cases (0.13%) with these diagnoses. These figures are around one power of ten lower than our values, which, along with possible cultural differences in the use, duration of therapy and diagnosis documentation, may also be caused by methodological differences such as the limited number of diagnoses included, the exclusion of patients with potentially interacting drugs and of patients without blood testing. Colantonio et al. 44 chose an entirely different approach which, based on administrative data, also suggested an algorithm for statin intolerance and, depending on the definition, identified a statin intolerance of 1.0% or 5.2%. The results are in the range of our study, but due to methodological differences, it has to be assumed that other patients are reported. In contrast to our approach, in their definition of statin intolerance, Colantonio et al. combined several criteria as dose reduction, switch to ezetimibe, diagnosis of rhabdomyolysis or discontinuation of therapy, “antihyperlipidemic event” after dose reduction or discontinuation of therapy or a change between three or more statins.

As our analysis of the continuity of statin therapy showed, just under half of the patients were still on therapy after 365 days; an interruption of therapy was observed in 29% (54% if one‐time prescriptions are included). Data on the persistence of statin therapy have, like the estimates of the frequency of statin intolerance, a broad range.^45^, ^46^ Zhang et al. 47 also report that in their cohort of statin recipients, an interruption of therapy was observed in 53%; 4.7% of their study patients had an indication of myalgia/myopathy. In our study, this share was in the group of those who were not given continuous therapy and had a statin change, with 4.8% on a comparable scale.

NICE guideline 48 and expert recommendations speak of statin intolerance, even if a second statin—provided there are no signs of rhabdomyolysis—is not tolerated and there are corresponding diagnoses.^34^, ^35^ Other causes as well as interactions as triggers must be ruled out. A subsequent discontinuation of therapy suggests intolerance. Our analysis showed that in this population, SAM was documented in around 4.2%. The available data do not include any information on the reasons for the discontinuation of therapy.

Schulman et al. 49 chose a similar approach to us in their validation study. They identified, based on ICD‐9 diagnosis code, a SAM of 2.9% for patients with statins who had a change in therapy.

In our view, SAM or statin intolerance cannot necessarily be concluded from the discontinuation rates. The estimates for SAM that we calculated are conservative, since unreported dose reductions or even therapy interruptions—as specified in treatment recommendations—may occur without documentation of a diagnosis of SAM. Even a change in the statin does not necessarily have to be associated with SAM. This may also be caused by regulatory drug policies (feedback to physicians concerning their adherence to simvastatin and pravastatin as lead compounds) or through marketing measures of pharmaceutical companies (eg, introduction of new substances, combinations or generics).

### 4.1 Limitations

Some limitations must be kept in mind when interpreting the results. Due to the database, only prescriptions that are charged to the SHI are reported, not the actual ingestion by the patient. This means that the exposure time is a result of the prescription pattern, with assumptions of the daily dose (here, 1 defined daily dose) having to be made. Statin intolerance was limited in this study to SAM using ICD‐10 GM‐coded inpatient and outpatient “muscle diagnoses”. These diagnoses cannot be validated externally, eg, through chart review or laboratory data. Our analysis is based on a single citation of the diagnosis. Since this is not a chronic disease (the complaints should subside after discontinuation or reducing the dose of the statins), we abstained from additional internal validation steps, such as multiple citations or citation by other, different doctor groups.

To our knowledge, for doctors in private practices, there are no economic incentives for documentation of a diagnosis in this area; therefore, we rather assume a potential under‐reporting instead of over‐reporting. Authors who had the options for validation consider the administrative data for reporting statin intolerance suitable 37, 43, 49—however, it must be added as a qualifier that this evaluation cannot be automatically transferred to health systems in other countries. That the diagnoses that we included are in fact SAM can only be deduced from the temporal connection. The diagnoses interpreted as statin intolerance occur in incidental statin recipients for this first time after 2 years without a diagnosis or complications, so the chronological coincidence of newly introduced statin therapy and occurrence of a diagnosis suggests a causal connection. If the pattern of treatment that indicates intolerance of the statin also leads to an increase in the percentage of statin intolerance rates, that is, change and discontinuation, a connection is all the more likely. The fact that despite the occurrence of statin intolerance, the statin therapy is continued without a change, does not argue against this circumstantial evidence, since it is known that patients tolerate side effects such as myalgia in consultation with the doctor and, if necessary, with a dose reduction.

We cannot rule out, that we might overestimate SAM as we did not apply a control group design. Our study design followed clinical practice, that is, the co‐occurrence of incident myopathy together with incident statin use would be rated as SAM by physicians and patients in every day practice.

As a further limitation we have to mention that we did not analyze different dosages of the statins with regard to any dose effect relationship for myopathy. We lack information on the daily doses prescribed by the physician, which is a prerequisite for such an analysis. When evaluating the results, documentation customs and billing rules (eg, diagnosis documentation to justify a CK value measurement) must be taken into account. It must also be considered that muscle pain is perceived differently according to the individual, and even physicians tolerate a CK value increase in their patients to different degrees.

### 4.2 Strengths

The strengths of the study are that, as a database, the entire SHI population in Germany was made available for the first time. Based on Germany's population, only around 12% (insured privately or uninsured) were not included. An important advantage compared to clinical studies is that an unselected patient group can be observed in an everyday care setting. Without selection and recall bias and a nonresponder rate of 0%, all statin recipients can be investigated with reference to the population. Another special feature is that the documentation of the SAM was described for different populations according to their pattern of treatment and not only for patients with statin changes. Thus, it appears that the SAM frequency differs substantially.

## 5 CONCLUSION

Compared to studies that use only hospitalizations due to SAM, the results reported here, by including the outpatient sector, also include milder cases of muscle complaints and give a realistic idea of SAM under everyday conditions. Depending on the observed pattern of treatment, the shares found in the study are between 1.3% and 5.0% and thus certainly do not represent a rare event in everyday health care. On the one hand, the results show that a not insignificant share of patients tolerate the therapy despite myalgia, since the statin therapy was continued for these patients without interruptions and without changing to an alternative statin. On the other hand, for patients who do not tolerate statins but need them due to their cardiovascular risk profile, an alternative treatment strategy must be found. There are recommendations for approaches to this issue.^14^, ^29^

## FINANCIAL SUPPORT

The study was financed by an unrestricted grant to the PMV forschungsgruppe from Sanofi‐Aventis.

## DISCLOSURE

Franz‐Werner Dippel is employee of Sanofi Aventis. Peter Ihle and Ingrid Schubert have no competing interest to declare.

## AUTHORS’ CONTRIBUTORS

PI, IS, and FWD designed the study, PI managed and analyzed the data, PI and IS prepared the draft of the manuscript, FWD reviewed the paper and all authors approved the final version of the manuscript. IS had full access to all the data in the study and takes responsibility for the integrity of the data and the accuracy of the data analysis.
